# Supplementary material for: Systemic Bevacizumab for Recurrent Respiratory Papillomatosis: A Scoping Review from 2009 to 2022
Source: Children (Basel). 2022 Dec 26;10(1):54. doi: 10.3390/children10010054 (PMC9856545; doi:10.3390/children10010054)
Supplement: Supplementary file 1 [file children-10-00054-s001.zip › children-1858225-supplementary.pdf]

Anexe 1. Search Strategy

1.1. Search for MEDLINE (Pubmed):

((("bevacizumab"[MeSH Terms] OR "bevacizumab"[All Fields]) OR "bevacizumab s"[All Fields]) OR "systemic bevacizumab"[All Fields]) AND (("Recurrent respiratory papillomatosis"[Title/Abstract] **AND**	Bevacizumab
"Recurrent respiratory papillomatosis"[Title/Abstract] OR "Human papillomavirus 11"[All Fields]) OR "papilloma"[All Fields]	Recurrent Respiratory Papillomatosis

1.2. Search for LILACS (LILACS BVS search):

mh:(bevacizumab) or tw:(systemic bevacizumab) AND tw:(Recurrent respiratory papillomatosis)	Bevacizumab
tw:(Human papillomavirus 11) or mh:(papilloma) or tw:(papilomatosis recurrente respiratoria) or mh:(papiloma) or tw:(papillomavirus humano 11)	Recurrent Respiratory Papillomatosis

1.3. Search for EMBASE:

'bevacizumab'/mj or 'systemic bevacizumab':ab,ti **AND**	Bevacizumab
'Recurrent respiratory papillomatosis':ab,ti OR 'human papillomavirus 11':ab,ti or 'papilloma'/mj	Recurrent Respiratory Papillomatosis

1.4 Search for Global Health (Ovid):

bevacizumab or systemic bevacizumab **AND**	Bevacizumab

recurrent respiratory papillomatosis OR papillomatosis OR Human papillomavirus 11	Recurrent Respiratory Papillomatosis

1.5. Search for ClinicalTrials:

bevacizumab or systemic bevacizumab **AND**	Bevacizumab
recurrent respiratory papillomatosis OR papillomatosis OR Human papillomavirus 11	Recurrent Respiratory Papillomatosis
